# Genome-Wide Identification of Expansins in *Rubus chingii* and Profiling Analysis during Fruit Ripening and Softening

**DOI:** 10.3390/plants13030431

**Published:** 2024-02-01

**Authors:** Zhen Chen, Danwei Shen, Yujie Shi, Yiquan Chen, Honglian He, Junfeng Jiang, Fan Wang, Jingyong Jiang, Xiaoyan Wang, Xiaobai Li, Wei Zeng

**Affiliations:** 1 Zhejiang Provincial Key Laboratory of Plant Evolutionary Ecology and Conservation, School of Life Sciences, Taizhou University, Taizhou 318000, China; chenzh@tzc.edu.cn (Z.C.); cherrysdw@163.com (D.S.); shiyujie@tzc.edu.cn (Y.S.); wxy3470117@163.com (X.W.); 2 Institute of Horticulture, Taizhou Academy of Agricultural Sciences, Linhai 317000, China; jjy5971@163.com; 3 Institute of Horticulture, Zhejiang Academy of Agricultral Sciences, Hangzhou 310021, China

**Keywords:** expansins, plant cell wall, fruit ripening, fruit softening, *Rubus chingii*

## Abstract

Improving fruit size or weight, firmness, and shelf life is a major target for horticultural crop breeding. It is associated with the depolymerization and rearrangement of cell components, including pectin, hemicellulose, cellulose, and other structural (glyco)proteins. Expansins are structural proteins to loosen plant cell wall polysaccharides in a pH-dependent manner and play pivotal roles in the process of fruit development, ripening, and softening. *Rubus chingii* Hu, a unique Chinese red raspberry, is a prestigious pharmaceutical and nutraceutical dual-function food with great economic value. Thirty-three *RchEXPs* were predicted by genome-wide identification in this study, containing twenty-seven α-expansins (EXPAs), three β-expansins (EXPBs), one expansin-like A (EXPLA), and two expansin-like B (EXPLBs). Subsequently, molecular characteristics, gene structure and motif compositions, phylogenetic relationships, chromosomal location, collinearity, and regulatory elements were further profiled. Furthermore, transcriptome sequencing (RNA-seq) and real-time quantitative PCR assays of fruits from different developmental stages and lineages showed that the group of *RchEXPA5*, *RchEXPA7,* and *RchEXPA15* were synergistically involved in fruit expanding and ripening, while another group of *RchEXPA6* and *RchEXPA26* might be essential for fruit ripening and softening. They were regulated by both abscisic acid and ethylene and were collinear with phylogenetic relationships in the same group. Our new findings laid the molecular foundation for improving the fruit texture and shelf life of *R. chingii* medicinal and edible fruit.

## 1. Introduction

*Rubus chingii* Hu is an economically important Eastern Chinese raspberry with a prestigious pharmaceutical and nutraceutical dual function [1,2]. The unripe fruit harvested at the stages of big green (BG) or green-to-yellow (GY) has been used in traditional Chinese medicine for tonifying kidneys and eyesight from the ancient Qin and Han Dynasties [1,3]. It has been widely used as a key ingredient in some Chinese Proprietary Medicines, such as Wuzi Yanzong Pill, Guilu Bushen Pill, Shenbao Mixture, and others, or soaked in white wine for medicinal liquor in folk [1]. More than 235 chemical constituents, including terpenoids, flavonoids, phenolics, polysaccharides, and others, have been identified and isolated in recent years [4,5,6]. The pharmacological studies indicated the therapeutical effects of anti-inflammation, antioxidantion, antidiabetic, anti-thrombosis, anti-tumor, neuroprotective, and liver protection of *R. chingii* fruit and leaves [1,4,7,8,9,10,11]. Simultaneously, the ripe fruit of *R. chingii* is popular for pleasant fresh fruit, like raspberries and blackberries, and has rich nutrients, including amino acids, phenolic acids, vitamins C and PP, ellagitannins, aromatics, and minerals, such as magnesium, zinc, and potassium, and it is beneficial for human health [2,12,13]. In the past, the utilization of *R. chingii* mainly depended on wild resources. During the last decade, spurred by increasing consumption, the *R. chingii* industry flourished in East and South China. The planting area was about 1000 ha in China in 2017 and reached more than 17,000 ha in 2019 with the production of nearly 70 thousand tons of fresh immature and ripe fruits [2]. Nevertheless, the fresh red berry of *R. chingii* is very perishable with a short shelf life, although it is regarded as non-climacteric like other berries [14]. Furthermore, to meet the demands of higher yield, greater quality, and excellent health benefits, thousands of germplasms from wild habitats were domesticated and crossed to breed superior cultivars [15]. For economic yield, fruit size is one of the major characteristics of breeding. However, there is little knowledge on *R. chingii* fruit expansion and ripening in the literature to date. Therefore, unveiling the mechanism and vital genes for fruit enlargement and softening of the unique plant, *R. chingii*, can provide useful information for fruit development, ripening, softening, and molecular breeding. In the genomic age, genomic sequencing results have greatly facilitated the illumination of key genes for traits. The first genome of *Rubus* was reported in 2016 [16]. *R. chingii* genomic sequencing was also completed in 2021 [6]. Based on this, several vital genes, such as flavonol synthesis (FLS), dihydroflavonol 4-reductase (DFR), and MYB308, have been investigated by integrated multiple-omics techniques [17,18]. In this study, genome-wide identification and RNA-seq of key genes for fruit ripening and softening were performed.

Fruit enlarging, ripening, and softening processes are closely associated with plant cell wall loosening, degradation, extension, and reconstruction. These changes in the cell wall are attributed to the depolymerization, and hydrolyzation of pectin, hemicellulose, cellulose, and microfibrils, which are induced by a complex set of enzymes, such as pectate lyase (PL or Pel), pectin methyl esterase (PME), polygalacturonase (PG), cellulase (Cel), xyloglucan endotransglucosylase/hydrolase (XTH), and others [19,20]. In addition, non-enzymatic cell wall proteins like expansins (EXPs) are also essentially involved in cell wall loosening and rearrangements in an acid-dependent manner [21]. EXPs are a group of structural proteins. A typical expansin is composed of 250–275 amino acids (aa) and has two functional domains and a signal peptide (SP): (1) the N-terminal 20–30 aa is for an SP to possibly use transmembrane transport for secretion; (2) the N-terminal 120–135 aa contributes to the six-stranded double-psi beta-barrel (DPBB_1) domain (PF03330) with glycoside hydrolase (GH45)-like catalytic activity; and (3) C-terminal 90–120 aa residues contribute to the Pollen_allerg_1 domain (PF01357), named Expansin_C [22,23]. According to phylogenetic analysis, the EXP family members can be divided into four subfamilies: α-expansin (EXPA), β-expansin (EXPB), expansin-like A (EXLA or EXPLA), and expansin-like B (EXLB or EXPLB) [23]. They work in divergent ways by a process of molecular “creep” to loosen the cell wall. EXPA members may promote cellulose microfibrils separation and entrail movement by local dissociation and the slippage of xyloglucans on the cellulose surface, whereas EXPB members may work on other glycans, like xylan, or dissolve the middle lamella of stigma to facilitate pollen tube invasion [21,23]. However, little information is available regarding the action mechanism of EXLA and EXLB.

EXPs exert cell wall loosening activity during seed germination, root growth and development, stem elongation, leaf initiation and growth, stomatal movement, fruit ripening and softening, and abscission, as well as in responses to biotic and abiotic stresses [23,24]. There are thirty-six members in *Arabidopsis thaliana*, including twenty-six EXPA, six EXPB, three EXLA, and one EXLB [22]. AtEXPA1-promoted stomatal opening, AtEXPA2 accelerated germination, AtEXPA3 enhanced growth and enlarged leaves, and AtEXPA10 increased the sizes of plant cells, leaves, and stems. The overexpression of *AtEXPB1* and *AtEXPB5* plants have longer petioles and soft stigma, respectively [24]. There are 35 and 114 candidate genes in *Fragaria vesca* and *F. ananassa*, respectively. Among these, *FvEXPA9* (*FveEXP11*), *FvEXPA12* (*FveEXP14*), and *FvEXPA27* (*FveEXP33*) were highly expressed in fruit ripening stages [21,25]; *FaEXPA2*, *FaEXPA5*, *FaEXP88,* and *FaEXP114* were closely related with fruit softening [21]. In addition, FaEXP7 was regarded as a potential softening activator [20]. However, there is no report about expansins in *Rubus*, which greatly limits the comprehensive understanding of fruit development and softness for these attractive berries. In this study, we identified 33 expansins encoding genes from *R. chingii* and systematically analyzed the bioinformation of *RchEXPs* and their roles in fruit ripening and softening. The results provided valuable insights into the characterization and function of the expansin genes family in *R. chingii*, an important Chinese herb and red raspberry.

## 2. Results

### 2.1. Identification of Expansin Family Members in Rubus chingii

A total of 39 genes and their proteins were initially obtained by the HMM search. Among these, thirty proteins consisting of two conserved domains (DPBB_1 and Expansin_C domains) were selected as expansins candidates in *R. chingii*. In addition, proteins with one complete DPBB_1 domain, such as LG04.3299, LG04.3300, and LG04.3301, were also retained according to strawberry expansins [21,25]. Therefore, 33 *RchEXPs* were identified in this study (Table 1 and Table S1). Furthermore, expansin genes in its sister plants, red raspberry (*Rubus idaeus*) and black raspberry (*Rubus occidentalis*), were also extracted from the genomic data, and a similar number of members were obtained (Tables S1 and S2), indicating the similarity of the same *Rubus*. The amino acids of RchEXPs ranged from 125 aa (RchEXPA8, molecular weight MW = 12,970.17) to 482 aa (RchEXPA4, MW = 53,196.43) and the isoelectric point (pI) of most members (26/33) were greater than 7, indicating these members might be rich in alkaline amino acids. The prediction of the subcellular location showed that RchEXPs were all located on the cell wall.

**Figure 1:**
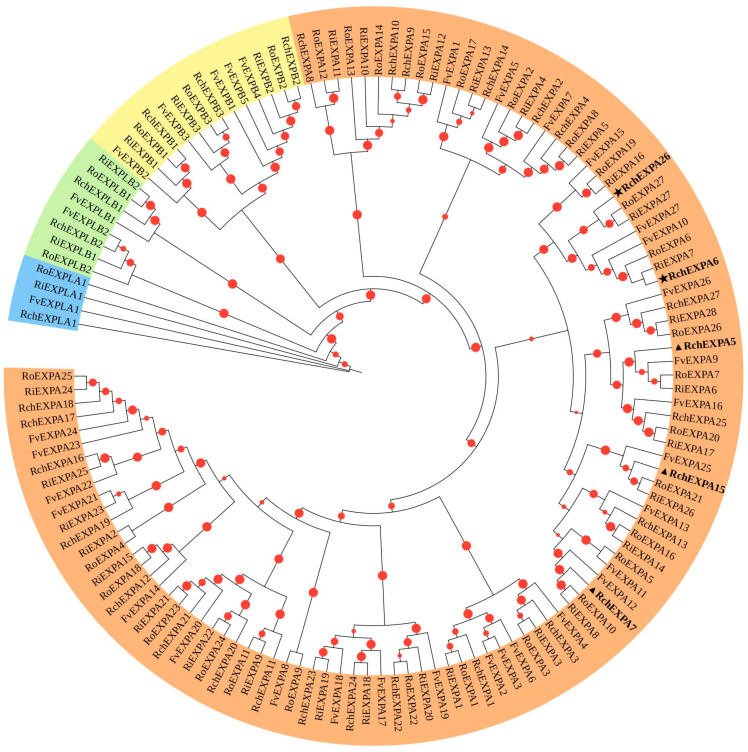
Phylogenetic tree of expansins from *Rubus chingii*, *Fragaria vesca*, *Rubus idaeus,* and *R. occidentalis.* EXPA, EXPB, EXPLA, and EXPLB subfamilies were presented in light orange, yellow, blue, and green. “★”indicates the same expression tendency during fruit development stages which was highest at the red stage, while “▲” indicates that these gene expressions were initiated in earlier stages of fruit.

**Figure 2:**
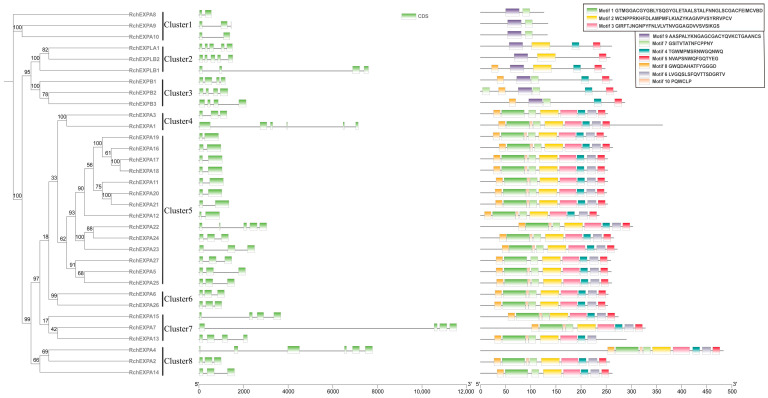
Phylogenetic relationships, gene structures of exon/intron, and motif compositions of expansins in *Rubus chingii*.

Phylogenetic analyses showed *RchEXPs* had relatively distant relatives with *AtEXPs* (Figure S1a), while they were more closely related to *FvEXPs*, *RiEXPs,* and *RoEXPs*, which belonged to the same Rosaceae (Figure 1). Based on the phylogenetic relationships with *FvEXPs*, all *RchEXPs* were divided into four subfamilies, including twenty-seven (81.8%) members of EXPA, three members of EXPB, one member of EXPLA, and two members of EXPLB (Table 1 and Table S1). Twenty-three orthologous pairs in *R. chingii* and *F. vesca*, and five paralogous pairs in *R. chingii*, were identified based on the high bootstrap values (>90%) (Figure 2 and Figure S1b).

### 2.2. Gene Structures and Protein Motifs of RchEXPs

The analyses of gene structures of *RchEXPs* exhibited the members of exons and varied from two to six, and introns varied from one to five (Figure 2). All *RchEXPBs* and *RchEXPLB1* had four exons, whereas *RchEXPLA* and *RchEXPLB2* contained five exons. Among the *EXPA* subfamily, 37% of the members contained two exons and 40.7% of the members contained three exons, whereas the others had four, five, or six exons. They could be divided into six clusters, and most members in the same cluster showed consistent exon numbers. All these results indicated the similarities of the same subfamily in structure.

Meanwhile, we provided the organization of the conserved motifs in Figure 2. Most members (77.8%) of the *EXPA* subfamily in *R. chingii* were composed of nine motifs, including Motifs 8, 1, 10, 7, 2, 3, 4, 6, and 5, whereas *RchEXPA3*, *RchEXPA14*, and *RchEXPA27* contained the above eight motifs, except for Motif 10. Unexpectedly, *RchEXPA8*–*10* contained only Motifs 9 and 7. *RchEXPBs* consisted of Motifs 8, 9, 7, 4, and 5. Motif 7 was found in all *EXPAs* and *EXPBs* (90.9% of the total) in *R. chingii,* except for *EXPLA* and *EXPLBs*. Oppositely, Motif 9 was not founded in most *RchEXPAs,* except for *RchEXPA8*–*10*. In addition, Motif 2 was not founded in *RchEXPBs*. All these results suggested the conserved structural evolution and consistency of the same subfamily.

### 2.3. Chromosomal Location and Synteny Analyses of RchEXPs

**Figure 3:**
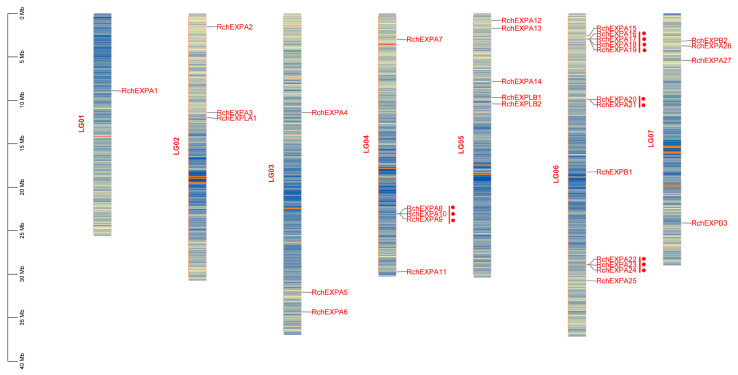
Chromosomal location of expansin encoding genes in *R. chingii.* The red dot means the tandem duplication events, including *RchEXPA8*–*10, RchEXPA16*–*19*, *RchEXPA20*–*21,* and *RchEXPA22*–*24*.

**Figure 4:**
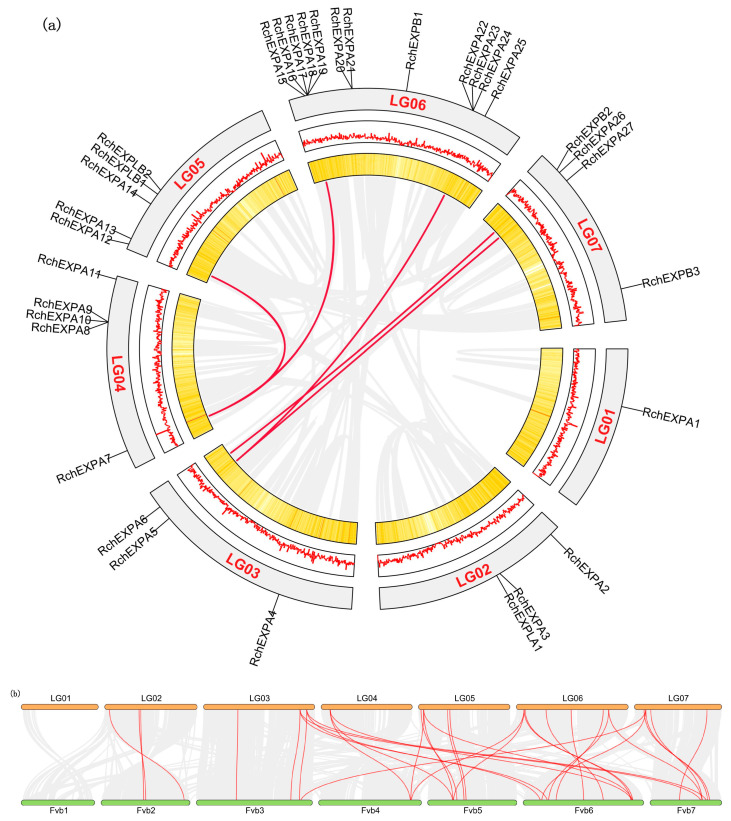
Synteny analyses of *R. chingii* and *F. vesa* genomes. (**a**) Synteny analysis of *R. chingii* genome. The gene density was displayed in the form of heat maps and lines. Syntenic blocks were linked by gray lines, and syntenic relationships of *EXP* members were highlighted by red color. (**b**) Syntenic relationships of *R. chingii* and *F. vesa* genomes. Syntenic *EXP* gene pairs between *R. chingii* and *F. vesa* were highlighted by red color.

*RchEXPs* were unevenly located on the seven chromosomes (Figure 3). The distribution pattern was similar to *F. vesca* [25]. Chromosome 6 (LG06) possessed the largest number (12/33) of RchEXPs encoding genes, including eleven members of the EXPA subfamily and one EXPB. Among these, three tandem duplication events occurred, including *RchEXPA16*–*19*, *RchEXPA20*–*21,* and *RchEXPA22*–*24*. Another tandem duplication was observed among *RchEXPA8*, *A9,* and *A10* in chromosome 4 (LG04). Chromosome 1 (LG01) had only one expansin gene (*RchEXPA1*). Chromosomes 2 (LG02) and 3 (LG03) both contained three *RchEXP* genes, whereas Chromosomes 4 and 5 (LG05) contained five *RchEXP* genes. *RchEXPBs* were distributed on chromosomes 5 and 7 (LG07). *RchEXPLA* was located on chromosome 2, whereas *RchEXPLBs* were located on chromosome 5, and both were in line with *F. vesca*. All these results supported the conserved evolution of expansins in berries, like strawberries and *Rubus*. Furthermore, synteny analysis indicated that only five collinear EXP gene pairs were found in *R. chingii*, including *RchEXPA6* and *RchEXPA26*, *RchEXPA5* and *RchEXPA27*, *RchEXPA5* and *RchEXPA25*, *RchEXPA7* and *RchEXPA13*, and *RchEXPA7* and *RchEXPA15* (Figure 4a). In addition, 33 collinear EXP gene pairs were identified between *R. chingii* and *F. vesca* (Figure 4b). Interestingly, the collinear events mainly occurred in *RchEXPA5*, *RchEXPA6*, *RchEXPA7*, *RchEXPA13*, *RchEXPA15,* and *RchEXPA26*. They all had three syntenic relationships with corresponding expansin genes in wild strawberry *F. vesca* (Table S3). For example, both *RchEXPA6* and *RchEXPA26* had collinear relationships with *FvEXPA10*, *FvEXPA15,* and *FvEXPA27*; both *RchEXPA7* and *RchEXPA25* had collinear relationships with *FvEXPA12*, *FvEXPA13,* and *FvEXPA25*; while *RchEXPA5* had collinear relationships with *FvEXPA9*, *FvEXPA16,* and *FvEXPA26* (Figure 4b and Table S3). All these results suggested that the orthologous and paralog gene pairs and roles of *EXPs* are found in berry fruit.

### 2.4. Cis-Regulatory Elements and Interaction Analyses of RchEXPs

**Figure 5:**
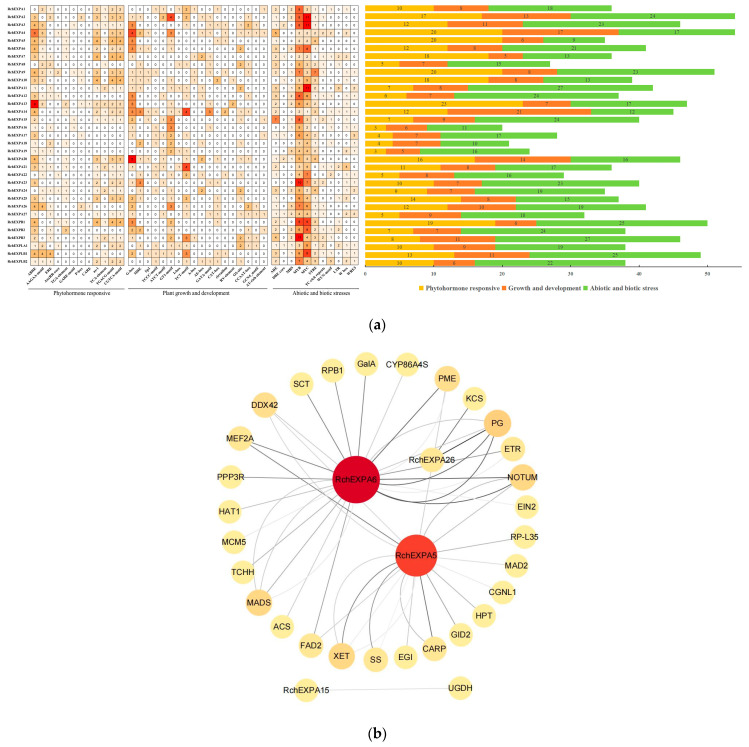
Predicted cis-acting elements and protein–protein interaction networks of expansins in *R. chingii*. (**a**) Predicted cis-acting elements and their numbers in the promoters of expansin genes in *R. chingii*. (**b**) Construction of protein–protein interaction networks of expansins and other proteins in *R. chingii*.

**Figure 6:**
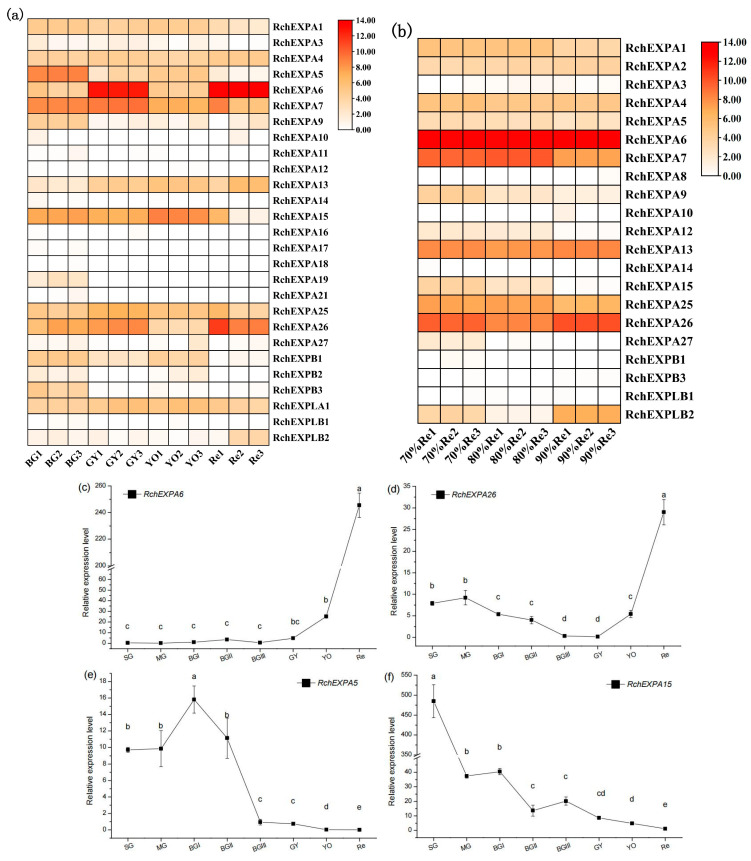
Expression profile of *RchEXPs* during fruit ripening and softening. (**a**) *RchEXPs* expression levels at the four representative stages, BGI, GY, YO, and Re, by RNA-seq. (**b**) *RchEXPs* expression levels at the red stages of the different degrees of ripeness by RNA-seq. (**c**–**f**) qPCR of *RchEXPA6*, *RchEXPA26*, *RchEXPA5,* and *RchEXPA15*. Different lower cases mean the significant difference at *p* < 0.05 by a one-way analysis of variance with the LSD method.

Analysis of cis-acting elements on the promoter sequences (2 kb upstream of ATG) could predict the regulatory factors and temporal–spatial expression patterns of *RchEXPs* (Figure 5a). First, cis-regulatory elements of a phytohormone response, such as abscisic acid-responsive elements (ABREs and AAGAA-motif), ethylene-responsive elements (EREs), auxin-responsive elements (AuxRR-core and TGA-element), gibberellin (GA)-responsive elements (GARE-motif, P-box and CARE), salicylic acid (SA)-responsive element (TCA-element) SA, auxin-response element (as-1), and jasmonic acid methyl ester (MeJA) responsive elements (TGACG-motif and CGTCA-motif) were detected in this study. The results showed promoters of most *RchEXPs* possessed ABRE sites, except for *EXPA1*, *A8*, *A22,* and *A24*. Moreover, the majority of them contained multiple ABRE sites, such as nine sites of the *RchEXPA13* promoter, six sites of the *RchEXPA4* promoter, and four sites of the *RchEXPA5*, *RchEXPA6*, *RchEXPA26*, *RchEXPA3*, *RchEXPA9*, *RchEXPA14*, *RchEXPA20*, *RchEXPB1,* and *RchEXPLB1* promoters. In addition, promoters of major *RchEXPs* had 1–4 AAGAA-motif sites. Both ABRE and AAGAA-motif sites indicated the possible ABA regulation of *RchEXPs*. Promoters of some *RchEXPs* had ERE sites, such as *RchEXPA26*, *RchEXPA1*–*2*, *RchEXPA7*–*9*, *RchEXPA11*–*12*, and others. There was an auxin-responsive element (TGA-element) in the promoter of *RchEXPA5*, *RchEXPA6,* and *RchEXPA7*. Most *RchEXPs* were associated with SA and MeJA, but may not respond to GA. Taken together, the five genes that were highly expressed in fruit (Figure 6), including *RchEXPA5*, *RchEXPA6*, *RchEXPA7*, *RchEXPA15,* and *RchEXPA26*, all could respond to the ABA signal, while only *RchEXPA7* and *RchEXPA26* were putatively regulated by Eth (Figure 5a). These results predicted the main regulation of ABA in non-climacteric fruits [14].

Secondly, cis-regulatory elements of plant growth and development were analyzed, including elements involved in light responsiveness (MRE, Sp1, TCCC-motif, ATCT-motif, GT1-motif, I-box, TCT-motif, A-box, AE-box and GATA-motif), meristem expression (CAT-box), circadian control (circadian), seed-specific regulation (RY-element), zein metabolism regulation (O2-site), endosperm expression (GCN4_motif), and MYBHv1 and AT-rich DNA binding protein (ATBP-1) binding sites (CCAAT-box and AT-rich element). The results indicated the action of *RchEXPs* was mainly regulated by light in divergent ways (Figure 5a). Finally, *RchEXPs* might actively participate in stress responses because of the most abundant sites in their promoters, including cis-acting elements of STRE (wound-responsive element), ARE (for anaerobic induction), LTR (involved in low-temperature responsiveness), MBS (involved in drought inducibility), TC-rich repeats (involved in defense and stress responsiveness), WUN-motif (wound-responsive element), DRE core, W box, and WRE3, especially MYB and MYC. Five genes, *RchEXPA5*, *RchEXPA6*, *RchEXPA7*, *RchEXPA15,* and *RchEXPA26,* all could respond to anaerobic and wound stress and could be regulated by MYBs and MYCs, whereas *RchEXPA6*, *RchEXPA15,* and *RchEXPA26* could respond to drought (Figure 5a).

Protein–protein interaction networks (PPIs) were constructed by a cytoscape tool based on transcriptome data (Figure 5b). RchEXPA6 was predicted to strongly interact with cell wall hydrolases, including PME and PG. It also could interact with an ethylene receptor (ETR) and ethylene-insensitive protein 2 (EIN2) and then result in ethylene-induced responses. Meanwhile, RchEXPA26 also could tightly interact with PG. These results suggest that *RchEXPA6* and *RchEXPA26* were apt in their involvement in fruit softening. RchEXPA5 was predicted to interact with PME, xyloglucan/xyloglucosyl transferase (XET), sucrose synthase (SS), and others. Hence, it might work earlier in fruit development. No PPI was found between RchEXPA7 and other proteins. Only UDP glucose 6-dehydrogenase (UGDH) was found to interact with RchEXPA15.

### 2.5. Expression Profiles of RchEXPs in Different Fruit Ripening and Softening Stages of R. chingii

**Figure 7:**
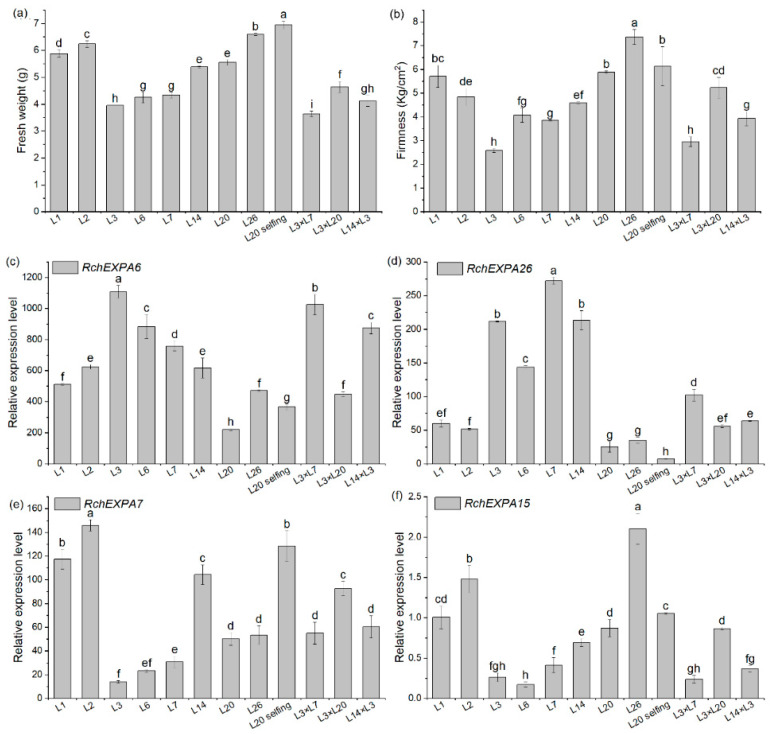

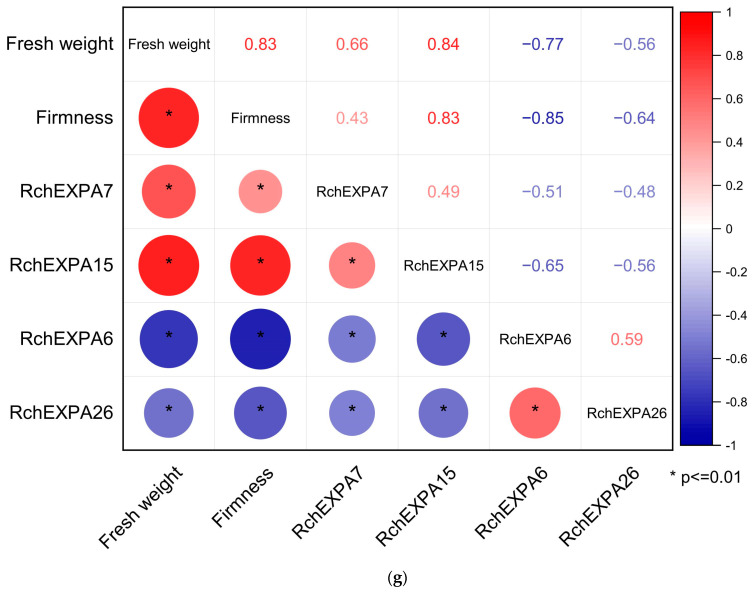
Biomass and real-time quantitative PCR of *RchEXPs* in red fruit of different *R. chingii* lineages. (**a**) Fresh weight (Fw); (**b**) fruit firmness; (**c**–**f**) qPCR of *RchEXPA6*, *RchEXPA26*, *RchEXPA7,* and *RchEXPA15.* Different lower cases mean a significant difference at *p* < 0.05 by a one-way analysis of variance with the LSD method. (**g**) Pearson correlation analysis of *RchEXPs* and fruit fresh weight and firmness based on the qPCR data of red fruits from different lineages. “*” means a significant difference at *p* ≤ 0.05.

The fresh weight of the eight stages (SG—small green, MG—middle green, BGI—big green I, BGII—big green II, BGIII—big green III, GY—green to yellow, YO—yellow to orange, Re—red) during *R. chingii* fruit development were 0.25, 0.48, 0.97, 1.02, 1.02, 1.43, 1.80, and 4.54 g, respectively [5]. Fruit firmness before the 60% red stages was all >15 kg/cm^2^, whereas fruit firmness of the 70% red, 80% red, and 90% red stages were 10.81, 8.47, 6.48 kg/cm^2^, respectively, indicating that the firmness was gradually reduced with fruit ripening. The data of transcriptomic sequencing showed the divergent expression pattern of all the expansin genes in *R. chingii*. Most *RchEXPs* were not detected or had very low expression levels in fruit, such as *RchEXPA2*–*3*, *RchEXPA8*, *RchEXPA10*–*12*, *RchEXPA14*, *RchEXPA16*–*24*, *RchEXPA27*, *RchEXPB2,* and *RchEXPLB1*. They might play vital roles in the root, stem, leaf, and flowers, or respond to different kinds of stresses. Nevertheless, some *RchEXPs* exhibited remarkable effects on fruit ripening and softening (Figure 6). Especially, *RchEXPA6* and *RchEXPA26* might be notably involved in fruit expanding and softening. The expression level of *RchEXPA6* at the immature BGI stage was 25.66 (FPKM, fragments per kilobase per million mapped fragments), while it rapidly increased to 6774.05 at the turning stage of GY, decreased at the YO stage, and then finally dramatically elevated to 14360.67 at the red stage, which was 559.65-fold in comparison with BGI (Figure 6a). The qPCR result verified its strong expression in the red stage (Figure 6c). The expression levels of *RcEXPA6* were related to fruit size, fresh weight, and firmness throughout the whole fruit development. In addition, *RchEXPA6* kept the highest expression level through the softening process from 70% to 90% ripe (Figure 6b). Simultaneously, the expression pattern of *RchEXPA26* was in line with *RchEXPA6* (Figure 6a–d). Oppositely, the expression levels of *RchEXPA5* and *RchEXPA15* were at high values at the beginning of the fruit set and earlier stages and decreased at later stages (Figure 6a,b,e,f). The FPKM of *RchEXPA5* and *RchEXPA15* at the BGI stage were 348.06 (449.34/1.29) and 146.04 (186.93/1.28) folds in comparison with the red stage, respectively. The *RchEXPA7* had relatively high expression levels from BG to 80% Re and then sharply reduced to a low level at 90% Re. Therefore, *RchEXPA5*, *RchEXPA7,* and *RchEXPA15* mainly participated in the process of cell expansion and fruit ripening. Therefore, red fruit from different lineages with different fruit sizes and different hardness were further analyzed. The fresh weights and firmness of L1, L2, L3, L6, L7, L14, L20, L26, L20⊗ (selfing), L3 × L7, L3 × L20, and L14 × L3 were measured (Figure 7a,b). The fruit of L3 × L7 was the smallest, and the fruit of L20⊗ was the biggest (Figure 7a). The 90% red fruit of L26, L20⊗, L1, L3 × L20, and L2 were relative hard, while the fruit of L3 × L7, L3, L6, L7, and L14 × L3 were softer (Figure 7b). The expression levels of *RchEXP6* and *RchEXP26* were relatively higher in softer fruits, while the expression levels of *RchEXP7* and *RchEXP15* were relatively higher in bigger fruits (Figure 7c–f). Pearson correlation analysis showed that *RchEXP6* and *RchEXP26* had significantly negative correlations with hardness, while *RchEXP7* and *RchEXP15* positively correlated with fresh weight (Figure 7g). These results verified the essential functions of *RchEXPA6* and *RchEXPA26* for fruit expanding and softening, while *RchEXP7* and *RchEXP15* mainly participated in fruit expansion and ripening.

These experimental results were greatly consistent with the bioinformatics analysis. In Figure 2, the phylogenetic relationships confirmed that *RchEXPA6* and *RchEXPA26* were in cluster 6, and this cluster was composed of only these two proteins. Moreover, in Figure 4, syntenic relationships also predicted the collinear and paralog gene pairs of *RchEXPA6* and *RchEXPA26*, suggesting the synergistic and complementary of the two EXPs. Simultaneously, *RchEXPA7* and *RchEXPA15* were in cluster 7, whereas RchEXPA5 was in cluster 5. In Figure 1, these three proteins were gathered in the same upper cluster. *RchEXPA7* and *RchEXPA15* showed a syntenic relationship. PPI analysis showed the interaction among RchEXPA6, RchEXPA26, PME, PG, ETR, and EIN2 (Figure 5b). The consistent functions of the two expansin genes *RchEXPA6* and *RchEXPA26*, or the three expansin genes *RchEXPA5*, *RchEXPA7,* and *RchEXPA15*, were first identified in this study. Unveiling the action mechanism and regulatory pathways of these vital *EXPs* would be vital for genetic modification and biotechnological breeding for fruit quality improvement and softening delay.

## 3. Discussion

The plant cell wall is pivotal for cell size, shape, and strength, and affects cell division and differentiation, growth rate, and functional realization [23,26]. It can be hydrolyzed, modified, or reconstructed by a series of enzymic and non-enzymic proteins. Expansins (EXPs) are noncatalytic structure proteins that are predominately located in the cell wall. They are involved in almost all aspects of plant growth and development, from germination to fruiting, by loosening the cell walls [22,24,25,27]. They also play vital roles in stress responses [22,28,29]. In total, 35 and 114 EXPs have been identified in woodland strawberry *Fragaria vesa* and octoploid-cultivated strawberry *F. ananassa* with distinct expression patterns [21,25]. In this study, we identified 33 *EXPs* from the *R. chingii* genome for the first time (Table 1, Figure 1, Figure 2 and Figure 3). Transcriptomic and quantitative PCR profiles verified that *RchEXPA5*, *RchEXPA7,* and *RchEXPA15* were synergistically involved in fruit expansion and ripening, while *RchEXPA6* and *RchEXPA26* might be essential for fruit ripening and softening (Figure 6). Our new findings laid a molecular foundation for improving fruit quality, controlling fruit firmness, and extending the storage life of *R. chingii* edible red fruit.

The expansins can be divided into four subfamilies according to the phylogenetic analysis. EXPA members are the main components of expansins in all kinds of plants, while EXPB members might mainly exist in Gramineae monocotyledons. In dicotyledonous plants, *Arabidopsis* has twenty-six, six, three, and one members of EXPA, EXPB, EXLA, and EXLB, respectively; polar (*Populus*) contains twenty-seven, three, two, and four members of the four subfamilies, respectively; and *F. vesa* consists of twenty-seven EXPAs, five EXPBs, one EXPLA, and two EXPLBs, respectively [25]. The proportion of EXPs in *R. chingii* is similar to *F. vesa. RchEXPs* are composed of twenty-seven EXPAs, three EXPBs, one EXPLA, and two EXPLBs (Table 1, Figure 2). These results suggest that the EXPA subfamily in dicotyledons might be expanded far more than the other three subfamilies. Nevertheless, Gramineae plants contain higher numbers of EXPB members. There are 34 EXPAs and 19 EXPBs in rice, 36 EXPAs and 48 EXPBs in maize, and 45 EXPAs and 29 EXPBs in moso bamboo [22,25]. The cell wall is fundamentally made up of different groups of polysaccharides, such as cellulose, hemicellulose, and pectin. Loosening of the polysaccharides network is the direct cause of cell wall looseness and cell expansions [30]. Xyloglucan is the main hemicellulose in the primary wall of dicotyledonous and non-gramineous monocotyledonous plants, comprising up to 20% of the wall dry matter [31]. However, the content of xyloglucan in gramineous plants was very low. EXPAs promote movement and separation of microfibrils by means of molecular creep for the dissociation and slippage of xyloglucans, while EXPBs work on another glycan, maybe xylan [19,23,32]. Among the xylans, arabinoxylan (AX) mainly exists in the grain of gramineous plants, glucuronoarabinoxylan (GAX) exists in vegetative tissues of gramineous plants and the primary cell wall of dicotyledonous, and glucuronoxylan (GX) mainly exists in the secondary wall of dicotyledonous and non-gramineous monocotyledonous plants [33]. Therefore, the various compositions of plant cell wall polysaccharides and the distinct action modes of EXPs might determine the proportion and numbers of EXP subfamilies.

Furthermore, it has been reported that xyloglucan disassembly might be an early event in fruit softening [24], and firmness is one of the decisive indexes of fruit quality and postharvest shelf life. The effects of expansins on fruit ripening and softening have been described in various horticultural plants. For example, the overexpression of tomato *Slexp1* hastened the softening process, while *Slexp1*-6 and *Slexp1*-7 mutants enhanced fruit firmness and could be stored for longer periods [34]. *FaEXP2* and *FaEXP5* were predominantly expressed in cultivated octaploid strawberry fruit, and the significant increase in the *FaEXP5* expression level was in close correlation with the rapid decrease in fruit firmness [19,35]. The above expression pattern of *EXP5* was also observed in Chilean strawberries [36]. In addition, *FaEXP7*, *FaEXP88,* and *FaEXP114* were also candidate genes for softening activation [20,21]. Our experiment evaluated the expression pattern of *RchEXPs* during fruit development and ripening and found that three expansin genes of *RchEXPA5*, *RchEXPA7,* and *RchEXPA15* were highly expressed at earlier stages but decreased at later stages, suggesting that they were predominantly involved in cell expansions and fruit ripening; while the expressions of the two expansin genes, *RchEXPA6* and *RchEXPA26*, were dramatically activated at red stage, indicating their essential role in fruit softening (Figure 6). In *F. vesa*, both Dong et al. [25] and Mu et al. [21] reported that three *FvEXPs* were highly expressed in ripening fruit. Among these, *FvEXPA9* (*FveEXP11*) and *FvEXPA12* had especially high expression levels in the turning stage, while *FvEXPA27* (*FveEXP33*) had especially high expression levels in the red stage and over-ripening stage. Phylogenetic analysis and synteny profiles showed that different expansins from diverse species belonging to the same clade or collinearity contributed to similar functions [21]. As expected, *RchEXPA6* had collinearity with *RchEXPA26* and *FvEXPA27* (Figure 4, Table S3), and the encoded protein had high structural similarity with FvEXP27 (Figure 2, Figure S1). Therefore, they exhibited the same function (Figure 6). Simultaneously, there were collinear relationships and close evolutionary relationships between *RchEXPA5* and *FvEXPA9*, *RchEXPA15* and *FvEXPA12*, as well as *RchEXPA15* and *RchEXPA7* (Figure 4), resulting in their consistent function. Overall, our results elucidated the key expansin encoding genes in fruit ripening and softening.

The regulator of expansins in *R. chingii* was also predicted through promoter and PPI analyses. Promoters of most *RchEXPs* showed multiple ABA-responsive element sites, and some of them also possessed Eth-responsive elements (Figure 5). Numerous studies have revealed that ethylene and ABA both could regulate fruit ripening in strawberries [14,37]. ERE was found upstream of *FvEXPA9* and *FvEXPA27* [25]. In this study, ERE was found in the promoter region of *RchEXPA7* and *RchEXPA26*, whereas it was absent in promoters of *RchEXPA5*, *RchEXPA6,* and *RchEXPA15* (Figure 5). Therefore, the ripening of *R. chingii* might be controlled by both ABA and Eth, and the RchEXPs act coordinately for fruit texture. It has been reported that the high relative expression of *FvEXP12* in the turning stage was attributed to the auxin-responsive element in its promoter [25]. Interestingly, *RchEXPA7* had collinearity with *FvEXP12* (Table S3), and *RchEXPA7* was also the orthologous gene for *FvEXP12*. Hence, the high expression level of *RchEXPA7* in the turning stage consisted of strawberry expansin.

In addition, many pieces of evidence also supported the effects of expansins on stress resistance. TaEXPA2 regulated by MYB transcription factor enhanced drought tolerance in wheat [29]. *PeEXPA19*, highly expressed in the leaves and roots of moso bamboo, had three MBS elements in its promoter and exhibited drought resistance, whereas PeEXPA44 showed downregulated expression under PEG stress [22]. Therefore, the functions of other expansins in *R. chingii* need to be further investigated. All expansins are distributed in different plant tissues and have distinct roles, whereas they are synergistically involved in plant growth, development, maturity, senescence, and response to stresses.

## 4. Conclusions

In conclusion, this study performed the first genome-wide identification of 33 expansins and their encoding genes in the unique Chinese raspberry, *Rubus chingii* Hu. The molecular characterization, biological function, and evolutionary pattern were elucidated. The results confirmed that three *RchEXPs*, including *RchEXP5*, *RchEXP7,* and *RchEXP15*, and two *RchEXPs*, *RchEXP6* and *RchEXP26*, played pivotal roles in fruit ripening and softening, respectively. Our study gives a comprehensive understanding of the expansin family in *R. chingii* and screens out the candidate genes for molecular breeding to slow down fruit softening and extend storage life.

## 5. Materials and Methods

### 5.1. Plant Material

*Rubus chingii* Hu seedlings were planted in our germplasm resource orchard (28°73′39″ N, 121°09′11″ E) for more than 5 years. Fruits from uniform 3-year seedlings of different superior lineages and crossing progenies were selected. For transcriptomic sequencing, every 20 fruits from the 4 representative stages, including BGI (big green I, 21 DPA, day-post-anthesis), GY (green-to-yellow, 42 DPA), YO (yellow-to-orange, 48 DAP), and the Re (red, 54 DPA, 90% ripe) of L7 (softer lineage with low firmness in red stages; other detailed characteristics of different lineages have been described in our previous work [2]) were harvested. Moreover, the ripening fruits of 70% red, 80% red, and 90% red from L26 (harder lineage with relatively high firmness) in red stages were also harvested for RNA-seq. For further real-time quantitative PCR assays, every 50 fruits in the eight stages from L7 were harvested in 2021, including SG (small green, 7 DPA), MG (medium green, 14 DPA), BGI, BGII (big green II, 28 DPA), BGIII (big green III, 35 DPA), GY, YO, and Re. Meanwhile, red fruit from different lineages were investigated, including L1, L2, L3, L6, L7, L14, L20, L26, L20⊗ (selfing), L3 × L7, L3 × L20, and L14 × L3. The fresh weight and fruit size were measured using electronic balance and vernier calipers. Firmness was determined using a digital durometer (FHT-05, Lantai, China).

### 5.2. Genome-Wide Identification of Expansin Family in R. chingii and Phylogenetic Analysis

The genome and protein sequences (v1.0) of *R. chingii* were obtained from the Genome Database for Rosaceae (GDR) (https://www.rosaceae.org/species/rubus/all) (accessed on 27 May 2021) [6]. In addition, *Rubus idaeus* Joan J Genome v2.0 (accessed on 15 September 2022), *Rubus occidentalis* whole genome assembly v3.0 (accessed on 30 April 2018), and *Fragaria vesca* Genome v4.0.a2 (accessed on 9 May 2019)were also downloaded from GDR. The PF03330 (DPBB_1 domain) and PF01357 (Expansin_C) domains were applied for the Hidden Markov Model (HMM) search with E-value < l0^−5^. The primary gene ID and protein sequences were extracted by TBtools, and then they were examined by the online tools in the Conserved Domain Database (CDD) (https://www.ncbi.nlm.nih.gov/Structure/bwrpsb/bwrpsb.cgi, accessed on 27 May 2021) and PFAM (https://pfam-legacy.xfam.org/search/sequence, accessed on 27 May 2021). Proteins with incomplete C or N terminal and incomplete domain DPPB_1 or Expansin_C were discarded. A maximum-likelihood phylogeny (ML-tree) of *R. chingii*, *R. idaeus*, *R. occidentalis*, *F. vesca,* and *A. thaliana* was constructed by TBtools and visualized by MEGA 11 and iTOL (https://itol.embl.de/, accessed on 27 May 2021). The *expansins* from the three *Rubus* species were renamed according to their chromosomal position and their similarity with *F. vesca*. The isoelectric point (pI) and molecular weight (MW) were calculated through ExPaSy (https://web.expasy.org/compute_pi/, accessed on 27 May 2021), and subcellular localization was predicted using online software on the Plant-mPLoc server (2.0 version) (http://www.csbio.sjtu.edu.cn/bioinf/plant-multi/#, accessed on 27 May 2021).

### 5.3. Motifs, Gene Structures, Chromosomal Location, and Synteny Analysis of Expansins in R. chingii

The gene structures of exon/intron were mapped using TBtools according to the genome annotation “*Rubus*_*chingii*_Hu.gff” file. Conserved motifs were identified with the online Multiple Em for Motif Elicitation (MEME, https://meme-suite.org/meme/tools/meme, accessed on 27 May 2021) tool with the parameter setting of the maximum number of motifs equal to 10. Gene density was profiled, and gene locations were visualized by TBtools. The synteny analysis in *R. chingii* genome and synteny analysis between *R. chingii* and *F. vesa* genomes were calculated with MCScanX (CPU for BlastP: 2; E-value: 1 × 10^−10^; Num of BlastHits:5) [38]. The syntenic diagram was visualized by an advanced circos and dual synteny plot using TBtools.

### 5.4. Cis-Regulatory Elements Analysis and Protein–Protein Interaction Network Construction of Expansins in R. chingii

The 2000 bp upstream genomic sequences of the expansin genes were extracted as the putative promoters to examine the cis-elements by PlantCARE (https://bioinformatics.psb.ugent.be/webtools/plantcare/html/, accessed on 27 May 2021). The predicted cis-acting elements and their numbers for phytohormone response, plant growth, and development, were summarized, respectively. The protein–protein interaction network (PPI) was analyzed based on transcriptome data, and the protein–protein interaction network (PPI) was constructed using cytoscape_v3.10.1.

### 5.5. Expression Analyses of RchExps during Developmental Stages

Transcriptomic sequencing of fruits from the four representative stages (BG, GY, YO, and Re) of L7 was carried out in 2017 using the Illumina (HiSeq X-Ten, San Diego, CA, USA) platform from the Beijing Genomics Institute (BGI, Wuhan, China). Approximately 536.05 Mb total clean reads with high quality (Q20 > 98.5%, Q30 > 82.5%) were obtained from all samples (4 stages in triplicate) [5]. Then, the de nova sequencing data were reanalyzed in 2021, according to the later published *R. chingii* genome [6]. The expression levels of *RchEXPs* were expressed by Fragments Per Kilobase per Million mapped fragments (FPKM) value (=10^6^ C/(NL/10^3^) and mapped as a heat map using TBtools. Moreover, RNA-seq of the ripening fruits of 70% red, 80% red, and 90% red from L26 was performed in 2021 using the DNBseq platform by the Beijing Genomics Institute (BGI, Wuhan, China). A total of 380.41 million total clean reads with high quality were obtained from all 9 samples (3 stages in triplicate), and the FPKM values of *RchEXPs* were extracted and mapped with the above method.

For a further real-time quantitative PCR (aPCR) assay, fruits of eight stages from L7 (SG, MG, BGI, BGII, BGIII, GY, YO, and Re) were harvested in 2021. A total of about 50 fruits at each stage were collected, and RNA was isolated using an OminiPlant RNA Kit (CW2598, CWBIO, Beijing, China). The cDNA synthesis by reverse transcription and qPCR determination were performed according to the previous methods [5]. The relative expression level was calculated using the 2^−∆∆Ct^ method. All the experiments were carried out in triplicate to quadruplicate, and the LSD test was selected for one-way analysis of variance using SPSS 26. Moreover, red fruits (90% ripe, edible) from different lineages, including L1, L2, L3, L6, L7, L14, L20, L26, L20⊗ (selfing), L3 × L7, L3 × L20, and L14 × L3, were harvested, and the qPCR of *RchEXP6*, *RchEXP7*, *RchEXP15,* and *RchEXP26* in these red fruits were determined with the above method.

## Figures and Tables

**Table 1:** Characteristics of RchEXP family members in *Rubus chingii* Hu.

Gene ID	Gene Name	Chromosome Location	Length (aa)	pI	MW	Subcellular Ocalization
LG01.812	RchEXPA1	LG01:8,877,139–8,884,215	361	6.99	39,910.71	Cell wall
LG02.327	RchEXPA2	LG02:1,544,766–1,543,798	256	8.86	27,373.94	Cell wall
LG02.2248	RchEXPA3	LG02:11,412,133–11,413,361	252	8.00	27,683.61	Cell wall
LG03.2086	RchEXPA4	LG03:11,399,062–11,406,813	482	9.30	53,196.43	Cell wall
LG03.4509	RchEXPA5	LG03:32,076,184–32,078,242	259	9.60	27,888.73	Cell wall
LG03.4886	RchEXPA6	LG03:34,333,208–34,334,320	253	8.12	27,197.39	Cell wall
LG04.560	RchEXPA7	LG04:2,963,719–2,975,241	327	8.83	35,055.07	Cell wall
LG04.3299	RchEXPA8	LG04:23,036,026–23,036,555	125	7.55	12,970.17	Cell wall
LG04.3300	RchEXPA9	LG04:23,053,077–23,054,497	133	5.03	13,924.83	Cell wall
LG04.3301	RchEXPA10	LG04:23,039,084–23,040,442	132	8.16	13,990.05	Cell wall
LG04.4234	RchEXPA11	LG04:29,721,537–29,722,605	252	7.52	27,668.67	Cell wall
LG05.148	RchEXPA12	LG05:803,994–804,890	235	9.02	26,237.85	Cell wall
LG05.336	RchEXPA13	LG05:1,698,742–1,700,885	289	9.49	31,609.97	Cell wall
LG05.1456	RchEXPA14	LG05:7,815,734–7,817,299	261	9.32	29,004.09	Cell wall
LG06.511	RchEXPA15	LG06:2,552,263–2,555,905	273	9.37	30,024.84	Cell wall
LG06.587	RchEXPA16	LG06:2,934,998–2,935,958	262	9.00	29,008.40	Cell wall
LG06.588	RchEXPA17	LG06:2,937,934–2,938,949	252	8.09	27,558.47	Cell wall
LG06.589	RchEXPA18	LG06:2,940,922–2,941,932	252	7.55	27,639.52	Cell wall
LG06.590	RchEXPA19	LG06:2,943,293–2,944,142	250	8.80	27,593.51	Cell wall
LG06.1815	RchEXPA20	LG06:9,876,023–9,877,025	250	8.01	26,551.37	Cell wall
LG06.1816	RchEXPA21	LG06:9,877,714–9,879,031	252	8.65	26,995.71	Cell wall
LG06.4033	RchEXPA22	LG06:28,894,710–28,897,718	302	9.07	33,583.85	Cell wall
LG06.4034	RchEXPA23	LG06:28,900,123–28,902,589	271	5.77	30,149.98	Cell wall
LG06.4035	RchEXPA24	LG06:28,905,229–28,906,521	264	8.75	29,355.39	Cell wall
LG06.4347	RchEXPA25	LG06:30,752,267–30,753,827	260	9.46	27,967.92	Cell wall
LG07.721	RchEXPA26	LG07:3,727,233–3,728,227	252	7.53	26,779.83	Cell wall
LG07.1027	RchEXPA27	LG07:5,399,695–5,401,139	258	9.01	27,605.21	Cell wall
LG06.2871	RchEXPB1	LG06:18,236,794–18,237,946	261	8.98	28,257.54	Cell wall
LG07.600	RchEXPB2	LG07:3,173,494–3,174,761	270	5.35	28,845.35	Cell wall
LG07.3409	RchEXPB3	LG07:24,111,895–24,113,983	286	4.39	30,073.51	Cell wall
LG02.2351	RchEXPLA1	LG02:11,988,434–11,989,907	260	8.45	28,204.14	Cell wall
LG05.1739	RchEXPLB1	LG05:9,646,051–9,653,623	247	6.87	27,091.51	Cell wall
LG05.1872	RchEXPLB2	LG05:10,406,187–10,407,678	257	4.88	28,062.50	Cell wall

## Data Availability

Data are contained within the article and supplementary materials.

## Supplementary Materials

The following supporting information can be downloaded at: https://www.mdpi.com/article/10.3390/plants13030431/s1.
