# Supplementary material for: Genome-Wide Identification of Expansins in Rubus chingii and Profiling Analysis during Fruit Ripening and Softening
Source: Plants (Basel). 2024 Feb 1;13(3):431. doi: 10.3390/plants13030431 (PMC10857257; doi:10.3390/plants13030431)
Supplement: Supplementary file 1 [file plants-13-00431-s001.zip › plants-2832667-supplementary.pdf]

Table S1 Numbers of expansin genes in 5 plant species

Species	EXPA	EXPB	EXPLA	EXPLB	Total	Reference
Arabidopsis thaliana	26	6	3	1	36	[23]
Fragaria vesca	27	5	1	2	35	[25]
Rubus chingii	27	3	1	2	33	Table 1
Rubus idaeus	28	3	1	2	34	Table S2
Rubus occidentalis	27	3	1	2	33	Table S2

Table S2 EXPs in *Rubus idaeus* and *Rubus occidentalis*

Gene ID	Name	Gene ID	Name
Rid.01g009060.m1	RiEXPA1	Ro01_G15922	RoEXPA1
Rid.02g041350.m1	RiEXPA2	Ro02_G02205	RoEXPA2
Rid.02g061740.m1	RiEXPLA1	Ro02_G15121	RoEXPA3
Rid.02g062800.m1	RiEXPA3	Ro02_G36059	RoEXPA4
Rid.02g084440.m1	RiEXPA4	Ro02_G18395	RoEXPLA1
Rid.03g110390.m1	RiEXPA5	Ro03_G08117	RoEXPA5
Rid.03g135100.m1	RiEXPA6	Ro03_G10614	RoEXPA6
Rid.03g139440.m1	RiEXPA7	Ro03_G12857	RoEXPA7
Rid.04g151750.m1	RiEXPA8	Ro03_G16418	RoEXPA8
Rid.04g176200.m1	RiEXPA9	Ro03_G32019	RoEXPA9
Rid.04g186810.m1	RiEXPA10	Ro04_G00051	RoEXPA10
Rid.04g186820.m1	RiEXPA11	Ro04_G21075	RoEXPA11
Rid.04g186830.m1	RiEXPA12	Ro04_G26476	RoEXPA12
Rid.05g215930.m1	RiEXPLB1	Ro04_G26477	RoEXPA13
Rid.05g217190.m1	RiEXPLB2	Ro04_G26480	RoEXPA14
Rid.05g220540.m1	RiEXPA13	Ro04_G27793	RoEXPA15
Rid.05g233440.m1	RiEXPA14	Ro05_G03265	RoEXPA16
Rid.05g235520.m1	RiEXPA15	Ro05_G14880	RoEXPA17
Rid.06g246900.m1	RiEXPA16	Ro05_G30510	RoEXPA18
Rid.06g250640.m1	RiEXPA17	Ro06_G05709	RoEXPA19
Rid.06g254050.m1	RiEXPA18	Ro05_G07225	RoEXPLB1
Rid.06g254060.m1	RiEXPA19	Ro06_G06114	RoEXPA20
Rid.06g254070.m1	RiEXPA20	Ro05_G21133	RoEXPLB2
Rid.06g265530.m1	RiEXPB1	Ro06_G17613	RoEXPA21
Rid.06g276990.m1	RiEXPA21	Ro06_G22018	RoEXPA22
Rid.06g277000.m1	RiEXPA22	Ro06_G28417	RoEXPA23
Rid.06g291400.m1	RiEXPA23	Ro06_G28418	RoEXPA24
Rid.06g291410.m1	RiEXPA24	Ro06_G20508	RoEXPB1

Rid.06g291420.m1	RiEXPA25	Ro06_G28680	RoEXPA25
Rid.06g292330.m1	RiEXPA26	Ro07_G19428	RoEXPA26
Rid.07g304890.m1	RiEXPB2	Ro07_G34601	RoEXPA27
Rid.07g306320.m1	RiEXPA27	Ro07_G12284	RoEXPB2
Rid.07g309410.m1	RiEXPA28	Ro07_G17391	RoEXPB3
Rid.07g335310.m1	RiEXPB3		

Table S3 Syntenic *EXP* gene pairs between *R. chingii* and *F. vesa.*

Gene name	Gene ID	link	Gene ID	Gene name
FvEXPA5	FvH4_2g38040.t1	—	LG02.327	RchEXPA2
FvEXPA4	FvH4_2g17030.t1	—	LG02.2248	RchEXPA3
FvEXLA1	FvH4_2g16110.t1	—	LG02.2351	RchEXPLA1
FvEXPA7	FvH4_3g20110.t1	—	LG03.2086	RchEXPA4
FvEXPA9	FvH4_3g36410.t1	—	LG03.4509	RchEXPA5
FvEXPA16	FvH4_6g13610.t1	—	LG03.4509	RchEXPA5
FvEXPA26	FvH4_7g22700.t1	—	LG03.4509	RchEXPA5
FvEXPA10	FvH4_3g40360.t1	—	LG03.4886	RchEXPA6
FvEXPA15	FvH4_6g10010.t1	—	LG03.4886	RchEXPA6
FvEXPA27	FvH4_7g25860.t1	—	LG03.4886	RchEXPA6
FvEXPA12	FvH4_4g31140.t1	—	LG04.560	RchEXPA7
FvEXPA13	FvH4_5g14760.t2	—	LG04.560	RchEXPA7
FvEXPA25	FvH4_6g48210.t3	—	LG04.560	RchEXPA7
FvEXPA14	FvH4_5g17040.t1	—	LG05.148	RchEXPA12
FvEXPA12	FvH4_4g31140.t1	—	LG05.336	RchEXPA13
FvEXPA13	FvH4_5g14760.t2	—	LG05.336	RchEXPA13
FvEXPA25	FvH4_6g48210.t3	—	LG05.336	RchEXPA13
FvEXPLB1	FvH4_5g20290.t1	—	LG05.1739	RchEXPLB1
FvEXPLB2	FvH4_5g21120.t1	—	LG05.1872	RchEXPLB2
FvEXPA12	FvH4_4g31140.t1	—	LG06.511	RchEXPA15
FvEXPA13	FvH4_5g14760.t2	—	LG06.511	RchEXPA15
FvEXPA25	FvH4_6g48210.t3	—	LG06.511	RchEXPA15
FvEXPA20	FvH4_6g33460.t1	—	LG06.1815	RchEXPA20
FvEXPA17	FvH4_6g16910.t1	—	LG06.4033	RchEXPA22
FvEXPA16	FvH4_6g13610.t1	—	LG06.4347	RchEXPA25
FvEXPA26	FvH4_7g22700.t1	—	LG06.4347	RchEXPA25
FvEXPB2	FvH4_6g26120.t1	—	LG06.2871	RchEXPB1
FvEXPA10	FvH4_3g40360.t1	—	LG07.721	RchEXPA26
FvEXPA15	FvH4_6g10010.t1	—	LG07.721	RchEXPA26
FvEXPA27	FvH4_7g25860.t1	—	LG07.721	RchEXPA26
FvEXPA26	FvH4_7g22700.t1	—	LG07.1027	RchEXPA27
FvEXPB4	FvH4_7g27130.t1	—	LG07.600	RchEXPB2
FvEXPB3	FvH4_7g10960.t1	—	LG07.3409	RchEXPB3

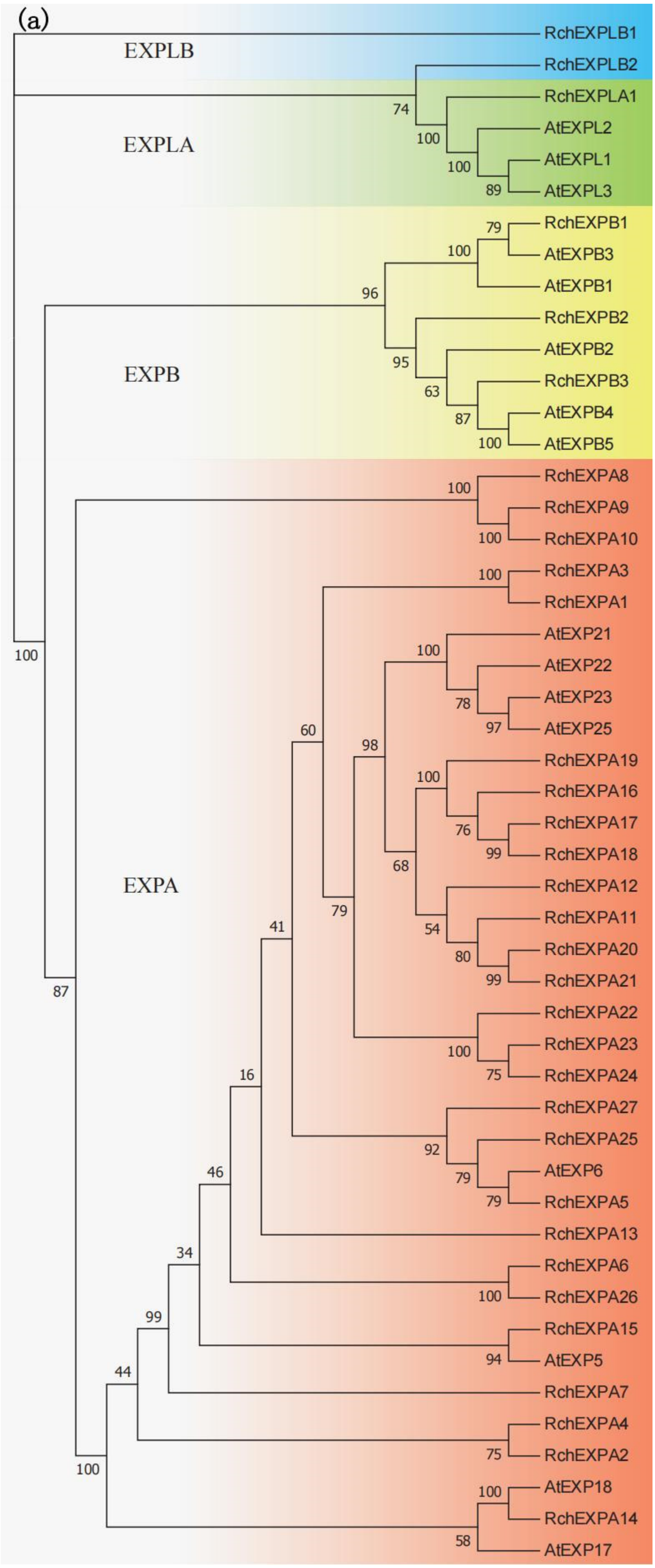
(a)
EXPLB
EXPLA
EXPB
EXPA
RchEXPLB1
RchEXPLB2
RchEXPLA1
AtEXPL2
AtEXPL1
AtEXPL3
RchEXPB1
AtEXPB3
AtEXPB1
RchEXPB2
AtEXPB2
RchEXPB3
AtEXPB4
AtEXPB5
RchEXPA8
RchEXPA9
RchEXPA10
RchEXPA3
RchEXPA1
AtEXP21
AtEXP22
AtEXP23
AtEXP25
RchEXPA19
RchEXPA16
RchEXPA17
RchEXPA18
RchEXPA12
RchEXPA11
RchEXPA20
RchEXPA21
RchEXPA22
RchEXPA23
RchEXPA24
RchEXPA27
RchEXPA25
AtEXP6
RchEXPA5
RchEXPA13
RchEXPA6
RchEXPA26
RchEXPA15
AtEXP5
RchEXPA7
RchEXPA4
RchEXPA2
AtEXP18
RchEXPA14
AtEXP17

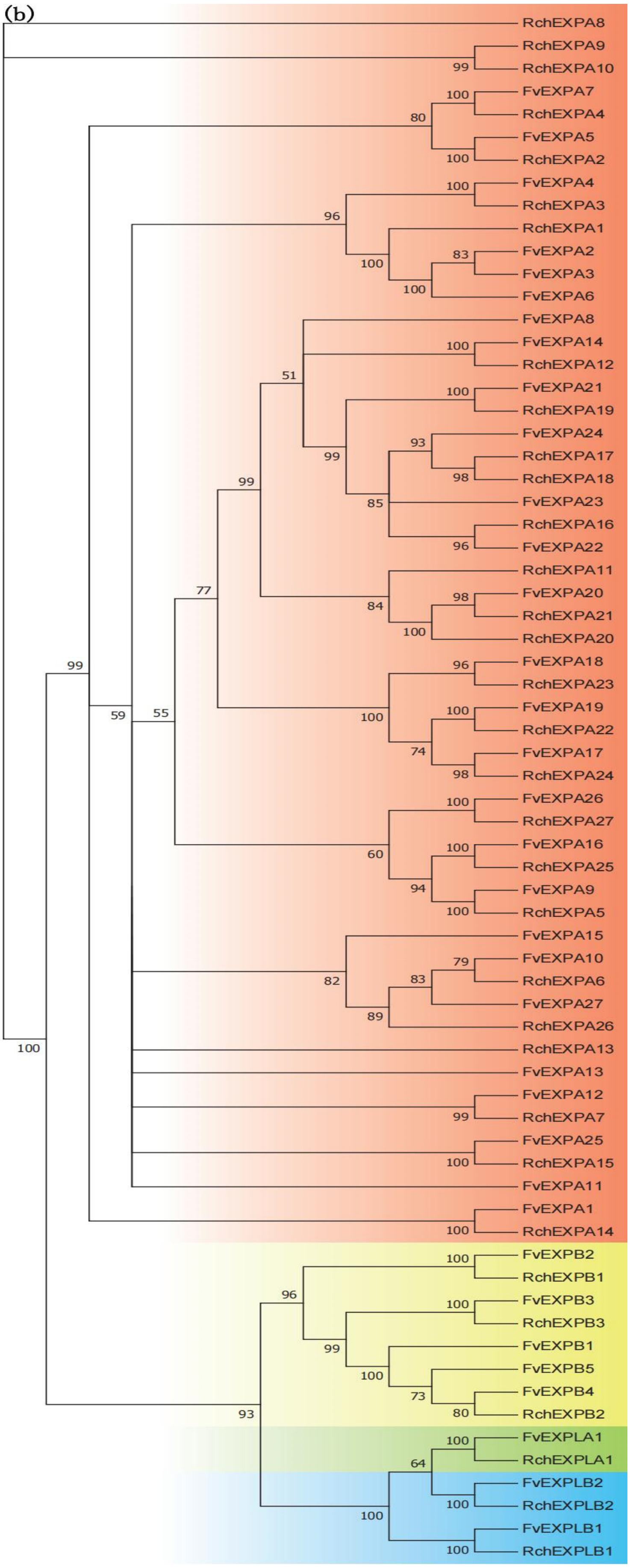

Figure S1 Phylogenetic analyses of expansins. (a) expansins of *Rubus chingii* and *Arabidopsis thaliana*; (b) expansins of *Rubus chingii* and *Fragaria vesca*
